# The complexity underlying invasiveness precludes the identification of invasive traits: A comparative study of invasive and non-invasive heterocarpic *Atriplex* congeners

**DOI:** 10.1371/journal.pone.0176455

**Published:** 2017-04-26

**Authors:** Jana Doudová, Jan Douda, Bohumil Mandák

**Affiliations:** 1 Department of Ecology, Faculty of Environmental Sciences, Czech University of Life Sciences, Prague, Czech Republic; 2 Department of Genetic Ecology, Institute of Botany, Průhonice, Czech Republic; Fudan University, CHINA

## Abstract

Heterocarpy enables species to effectively spread under unfavourable conditions by producing two or more types of fruit differing in ecological characteristics. Although it is frequent in annuals occupying disturbed habitats that are vulnerable to invasion, there is still a lack of congeneric studies addressing the importance of heterocarpy for species invasion success. We compared two pairs of heterocarpic *Atriplex* species, each of them comprising one invasive and one non-invasive non-native congener. In two common garden experiments, we (i) simulated the influence of different levels of nutrients and population density on plants grown from different types of fruits and examined several traits that are generally positively associated with invasion success, and (ii) grew plants in a replacement series experiment to evaluate resource partitioning between them and to compare their competitive ability. We found that specific functional traits or competitiveness of species cannot explain the invasiveness of *Atriplex* species, indicating that species invasiveness involves more complex interactions of traits that are important only in certain ecological contexts, i.e. in specific environmental conditions and only some habitats. Interestingly, species trait differences related to invasion success were found between plants growing from the ecologically most contrasting fruit types. We suggest that fruit types differing in ecological behaviour may be essential in the process of invasion or in the general spreading of heterocarpic species, as they either the maximize population growth (type C fruit) or enhance the chance of survival of new populations (type A fruit). Congeners offer the best available methodical framework for comparing traits among phylogenetically closely related invasive and non-invasive species. However, as indicated by our results, this approach is unlikely to reveal invasive traits because of the complexity underlying invasiveness.

## Introduction

The identification of species traits that promote invasiveness has for decades been one of the main challenges in invasion ecology [1–3]. This question is of great significance for the understanding of plant success in general and particularly the mechanisms of plant invasions [4,5]. It has been documented that invasive plant species, compared to their non-invasive congeners, generally attain greater values of life-history and functional traits, such as plant size, fecundity, growth rate, specific leaf area, photosynthetic capacity or survival (see [4,6] and references therein). However, contradictory results of global analyses searching for important traits such as plant size [4,7], seed mass [8,9] and plant fecundity [8,10] have been reported, confirming that it might be impossible to predict invasibility based on traits alone [9,11].

Another frequently addressed question is whether invasive species possess higher phenotypic plasticity than co-occurring natives [10,12,13]. Invasive plant species have repeatedly been shown to be more phenotypically plastic in a wide range of traits correlated with resource supply management [10,13–15], but most studies have failed to find an associated fitness benefit [13,16]. With respect to the vague results of the trait-based approach to predicting invasive potential, some authors have stated that the predictive power of traits is weak [11,17,18]. Species invasiveness clearly involves more complex interactions of traits that may be important only in certain ecological contexts. Therefore, one of the more targeted approaches how to improving the current knowledge about traits determining biological invasions is to perform more intensive studies on particular model systems of organisms [17].

The fluctuating resource hypothesis, postulated by Davis, Grime and Thompson [19], states that invasions are facilitated by high resource availability resulting from environmental disturbances. The hypothesis assumes that invading species are more successful in invaded communities, provided that they do not face intense competition for resources from resident species. Anthropogenic habitats characterized by higher availability of resources and frequent disturbances are known to be the most invaded [20]. One of the strategies plants use to spread effectively despite frequent disturbances is heterodiaspory (sensu [21,22]). The production of different types of diaspores with differing dispersal potential (e.g. with or without a pappus) and patterns of dormancy (i.e. nondormant vs dormant) by single individuals has been described across different families of angiosperms, but most frequently in the Asteraceae and Chenopodiaceae [21–23]. This behaviour allows annuals to escape from the harshness and unpredictability of their habitat in space (dispersal) and time (delayed germination via dormancy). While non-dormant seeds ensure immediate germination when conditions are optimal, at least a portion of dormant seeds remain in the seed bank. As concerns dispersal and dormancy, it is usually found that one morph has a high or relatively high dispersal ability and little or no dormancy, while the other one has a low dispersal ability and strong or relatively strong dormancy. This strategy has been described as a ‘bet-hedging’ adaptation to the unpredictability of desert environments [24,25]. Alternatively, it may allow plants to evade the negative effects of density [26], sib competition [27,28] or larval herbivory [29]. Since the early work of Venable and Lawlor [25], many aspects of heterodiaspory have been studied. It has been shown that heterodiasporic seeds or fruits significantly differ in germination capacity (e.g. [30,31]), dispersal [32–34], seed bank dynamics [35], dormancy patterns [36,37] and competitive abilities [38,39]. There is also evidence that plant allocation to different fruit types may be environmentally dependent [40–42], as it is likely an important adaptive strategy to the harsh and unpredictable desert environment [42–47].

Congener species represent an optimal model for detecting traits responsible for invasion success [48]. Key differences in traits can be revealed by comparative analyses of species with different invasive success if they are phylogenetically closely related. Two invasive heterocarpic *Atriplex* species (*A*. *sagittata* and *A*. *tatarica*), which have their non-invasive and non-native congeners (*A*. *hortensis* and *A*. *rosea*, respectively), grow in Central Europe [44]. *A*. *sagittata* and *A*. *hortensis* belong to the section Atriplex [49], which represents primitive oraches within the genus *Atriplex* [50]. Whereas *A*. *sagittata* is the most invasive representative of the genus in Central Europe, *A*. *hortensis* is a species with limited distribution that is strongly dependent on its relatively common cultivation and subsequent escapes from cultivation [43]. The section Sclerocalymma contains *A*. *tatarica* and *A*. *rosea* [49], which are evolutionarily derived species within the genus *Atriplex* with C_4_ photosynthesis [50]. *Atriplex tatarica* is a common species distributed mainly in the lowlands of Central Europe and has been expanding further in recent years [51]. On the other hand, *A*. *rosea* is a species that used to be relatively common in Central European villages; today, however, it remains only at a few localities [44]. The section Atriplex comprises species bearing three types of fruit (i.e. *A*. *sagittata*–*A*. *hortensis* group) with different patterns of dormancy and germinability both among fruit types and between species [43]. By contrast, species of the section Sclerocalymma produce only two types of fruit (i.e. the *A*. *tatarica*–*A*. *rosea* group) that are not too different in the level of dormancy and germination characteristics both overall and between species [43].

In this study, we compared two pairs of phylogenetically closely related heterocarpic congeners, *A*. *sagittata*–*A*. *hortensis* and *A*. *tatarica*–*A*. *rosea*, in two common garden experiments, hypothesising that (a) different traits explaining invasiveness may exist between different evolutionary lineages and that (b) heterocarpy is an important trait positively related to invasiveness. To test these hypotheses we carried out two experiments comparing (i) plants grown from different types of fruits under different levels of nutrients and population densities, focusing on traits that are generally positively associated with invasion success; and (ii) the competitiveness of species pairs vying for the same resources. By studying two pairs of closely related heterocarpic species with different patterns of dormancy and germinability, we addressed the following questions: (1) Are there any differences in traits between invasive and non-invasive *Atriplex* species that are consistent across evolutionary lineages? (2) If so, are these differences dependent on nutrient supply and neighbour density? (3) Are there any differences in traits between plants originating from different fruit types? (4) Do trait differences between plants growing from different fruit types contribute to invasion success? and (5) Does competitive ability explain invasiveness across evolutionary lineages?

## Materials and methods

### Ethics statement

The collections used for this study did not involve protected species, and no specific permissions were required for sampling activities in these locations.

### Study system

The genus *Atriplex* L. (family Amaranthaceae) comprises about 270 species, whose distribution centres are primarily deserts and semideserts of most continents [51]. Many of them are annuals that typically exhibit some kind of heterocarpy [21].

We compared two closely related invasive and non-invasive *Atriplex* species belonging to two different taxonomic groups (see Table 1 for a detailed overview of species characteristics). The groups differ especially in the number of heterocarpic fruits and the type of photosynthetic pathway. The more ancestral section Atriplex, represented by *A*. *sagittata* and *A*. *hortensis*, is characterized by three types of fruits and the C_3_ photosynthetic pathway. The evolutionarily more derived section Sclerocalymma includes the C_4_ species *A*. *tatarica* and *A*. *rosea*, which produce only two different types of fruit. The fruits differ both morphologically (mainly in colour and the presence/absence of bracteoles) as well as ecologically [43,44]: (1) The first fruit type (hereafter referred to as type A), present only in representatives of the section Atriplex, originates from female or bisexual ebracteate flowers. Fruits of this type are small, black and lens-shaped with a glossy, smooth testa and a 5-lobed perianth. (2) The second fruit type (type B) is produced by female bracteate flowers. It is intermediate in size and has a similar appearance to the previous type, but it is covered by extended bracteoles. (3) The third fruit type (type C), produced by female bracteate flowers, is rather large, brown and covered by extended bracteoles that are larger than those of type B fruits. The individual fruit types differ especially in their germinability rates and patterns of dormancy. While type A fruit is the most dormant and forms a persistent seed bank, type C fruit is non-dormant and ensures almost 100% germination in the first year after ripening. Type B fruits exhibit intermediate germination characteristics [43].

**Table 1:** Distribution and basic characteristics of species under study.

	Species			
	*A*. *sagittata* Borkh.	*A*. *hortensis* L.	*A*. *tatarica* L.	*A*. *rosea* L.
**Section** [53]	Atriplex L.	Atriplex L.	Sclerocalymma Aschers.	Sclerocalymma Aschers.
**Invasive status** [44,52]	Invasive	Non-invasive	Invasive	Non-invasive
**Native range** [53, 54,56,58,60]	Central Asia, Asia Minor and Eastern Europe	Probably cultivar of *A*. *sagittata*	Central Asia, Asia Minor, South-west Siberia, North Africa	Mediterranean
**Exotic range** [53, 54,56,58,60]	Central Europe, South Africa and North America	Cultivated in temperate zone of northern hemisphere, mainly in Europe and occasionally escaping from cultivation. Australia.	Western and Central Europe, North and South America	Western and Central Europe, South Africa, North and South America
**Life form** [58]	Annual	Annual	Annual	Annual
**Height** [58]	1.5 m	1.5 m	0.5 m	0.5 m
**Flowering** [58]	July–August	July–August	July–November	July–September
**Bracteole characteristic** [53,58]	Leaf like	Leaf like	Woody	Woody
**Number of fruit types** [58]	3	3	2	2
**Kranz anatomy** [55]	No	No	Yes	Yes
**Chromosome number** [57,59]	2n = 2x = 18	2n = 2x = 18	2n = 2x = 18	2n = 2x = 18

The data for individual species abundance are adopted from [44], i.e. casual—plants that reproduce occasionally in areas directly influenced by humans (e.g. common cultivation); invasive—plants produce offspring in large number and spreading quickly on the territory of the Czech Republic (invasive status is based on the concept of [52]). Data are based on the following literary sources: [53–60]

### General description of experiments

We compared two pairs of invasive and non-invasive species (*Atriplex sagittata* with *A*. *hortensis* and *A*. *tatarica* with *A*. *rosea*) in two common garden experiments. In the first experiment (hereafter referred to as the “heterocarpy experiment”), we compared species for several traits across different fertilization levels and population densities, and searched for trait differences among plants grown from different types of fruit. In the second experiment (hereafter referred to as the “replacement experiment”), we used the replacement series design to examine whether species belonging to the same group use the same resources to compete against each other and to compare their competitive ability.

Both experiments were conducted in an experimental garden at Průhonice in 2014, Central Bohemia, Czech Republic (49°59′41″ E, 14°33′56″ W). Fruits of all *Atriplex* species, i.e. *A*. *sagittata*, *A*. *hortensis*, *A*. *tatarica* and *A*. *rosea*, were collected in autumn 2013 in a waste ground in Prague, Czech Republic (50°07′27″ E, 14°30′29″ W). Bracts were removed from fruits, and fruits were sorted according to the species and type (A, B and C), and stored in paper bags at room temperature until use.

Seeds were sown into small plastic flats 7 cm (width) × 7 cm (length) × 9 cm (depth) and watered daily. Before sowing, all seeds were scarified (see [61]) to reach high germination percentages (see [43]). After 20 days, seedlings were transplanted into larger experimental pots 19 cm (width) × 19 cm (length) × 19 cm (depth) (6.9 L) in size. To reduce the variation in initial seedling size, only seedlings that germinated in the course of a single day (i.e. the day representing the peak of the population germination) were used for the experiments. Plants that died after being transplanted were replaced with ones of comparable size for the first two weeks of each study, after which no plants needed to be replaced.

### Heterocarpy experiment

We used a randomized block design with 8 replicates. Each block consisted of 24 pots containing *A*. *sagittata* together with *A*. *hortensis* (representing a combination of two species, three fruit types, two densities and two fertilization levels) and 16 pots containing *A*. *tatarica* together with *A*. *rosea* (representing a combination of two species, two fruit types, two densities and two fertilization levels).

The experiment consisted of (1) a single plant in the pot (low density**)** and (2) one target plant surrounded by four border plants; the plants in each pot had a regular spatial distribution, with the target plant located in the centre and border plants in the space between the centre and the pot corners (high density). The fertilization treatment included (1) watering with a Cererit solution alternated with pure water (low fertilization), (2) watering only by the Cererit solution (high fertilization). The pots were watered every three days, and the same amount of water/fertilizing solution was used for each pot (0.2 L). Sterilized sand subsequently fertilized depending on the treatment was used as the potting substrate. For fertilization, the complete fertilizer Cererit was used (N—11%, P_2_O_5_—9%, K_2_O—14%, MgO—1.5%, trace elements: B, Co, Cu, Mg, Mn, Zn).

We measured plant size (cm) four times per season (14 June, 30 June, 19 July and 25 August) to obtain a non-destructive estimate of the relative growth rate (RGR). The RGR was calculated as the linear-regression slope of ln(plant size). The experiment was run until all plants had matured and bore ripe fruits. This took 113 days, after which we dried and weighed each plant to determine fruit and stem mass (g).

### Replacement experiment

For our inter-specific competition experiment, only non-dormant type C fruits were used because of their high germinability [43] and high rate of seedling survival [47]. The competition experiment was set up in the de Wit [62] replacement series design, i.e. whilst the overall density is held constant, frequencies of each of the two species grown together are varied from 0 to 100%. To determine the constant final yield of individual species, monocultures of 1, 2, 4, 6 and 8 plant(s) per pot were planted. All plant species reached constant final yield at 4 to 8 plants per pot (Kruskall-Wallis test; *P* ≥ 0.262). The final density used in the replacement series was 8. Six replications were used per density. Five different frequencies of two species (*i*, *j*) were used: 0*i*:8*j*, 2*i*:6*j*, 4*i*:4*j*, 6*i*:2*j*, 8*i*:0*j*. Each mixture combination contained an invasive and non-invasive species of each phylogenetic group, i.e. *A*. *sagittata—A*. *hortensis* and *A*. *tatarica—A*. *rosea*. On the day the plants were harvested (after 119 days of the experiment), they were dried at 80°C for 48 hours, and the total mass of all plants was weighed separately.

Relative yield (*RY*) and relative yield total (*RYT*) were calculated for each species according to the following formulae [63,64]:

relative yield of species *i*: ${\text{RY}}_{i}=\frac{Y_{\text{ij}}}{Y_{\text{ii}}}$,
relative yield of species *j*: ${\text{RY}}_{j}=\frac{Y_{\text{ji}}}{Y_{\text{jj}}}$,
relative yield total: $\text{RYT}={\text{RY}}_{i}+{\text{RY}}_{j}$,
  where *Y*_*ij*_ or *Y*_*ji*_ is the total yield of species *i* (or *j*) when grown with species *j* (or *i*), and *Y*_*ii*_ (or *Y*_*jj*_) is mean total yield of species *i* (or *j*) in monoculture.

The gain or loss of biomass due to inter-specific competition was determined by calculating the aggressivity (*A*) of each species as follows [64]:

aggressivity of species *i*: $A_{i}=\frac{{\text{RY}}_{i}}{p}-\frac{{\text{RY}}_{j}}{q}$,
aggressivity of species *j*: $A_{j}=\frac{{\text{RY}}_{j}}{q}-\frac{{\text{RY}}_{i}}{p}$,
  where *p* and *q* are proportions of species *i* and *j*, respectively, in mixture (*p + q* = 1). An aggressive species will have a higher aggressivity index than a subordinate species [64,65].

**Fig 1:**
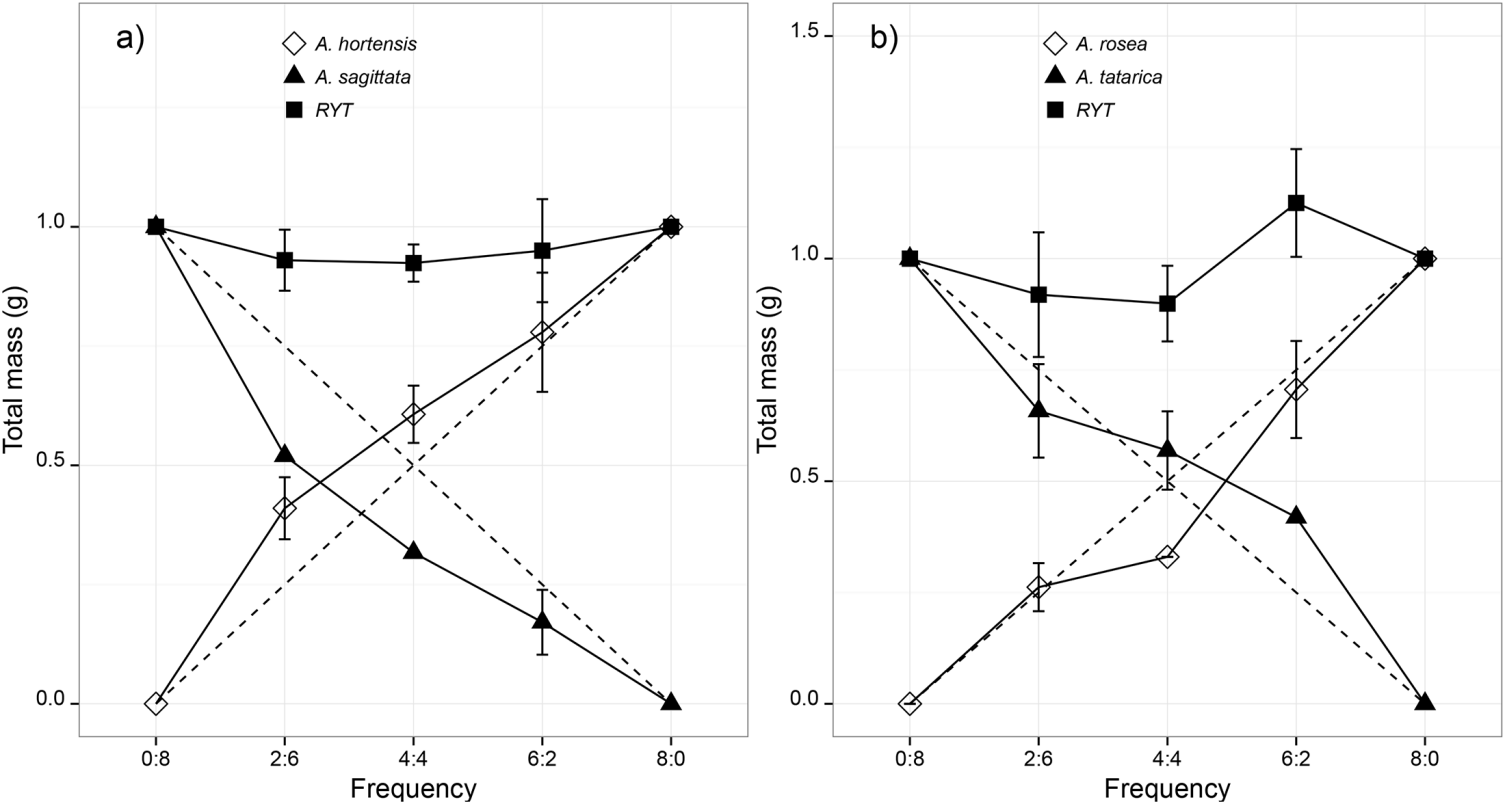
Results of the replacement experiment. Replacement series diagrams of two pairs of *Atriplex* congeners illustrating mean (± *SE*) relative yield and relative yield total as a function of species frequencies. The diagonal dashed lines are the expected relative yields when plants of species grow equally well in mixture and in monoculture. Standard error is shown if any difference from its expected value (*P* ≥ 0.05) was detected. Invasive species are indicated by black triangles and non-invasive ones by white diamonds; black squares indicate total relative yield.

Relative yields (*RY*) were then plotted into replacement diagrams against appropriate planting proportions. Comparisons of the actual *RY* of each species with their expected *RY* (the species grow equally well in monoculture and in mixture) are indicated by diagonal dashed lines in replacement diagrams (Fig 1). There are three possibilities, each indicating a different situation when the species are grown in mixture. (1) If the actual *RY* curve of one species is concave and that of the second is convex, then the species compete against each other; (2) if the actual *RY* curve of each species is convex, niche differentiation is indicated; and (3) if the actual *RY* curves of both species are concave, then we can assume mutual antagonism. The consequences of *RYT* values depend on whether they are equal to 1 (implying that there is competition), greater than 1 (implying niche differentiation), or lower than 1 (implying mutual antagonism) [63].

### Statistical analyses

In the heterocarpy experiment, total mass, fruit mass and relative growth rate (RGR) were analysed with generalized linear models including Section, Invasive(Pair), Fertilization, Density, Fruit type and all second-order interactions with the exception of the interaction Fertilization × Density. All factors were considered fixed. The effects of Block and all interactions with Block were tested, and as all were non-significant (*P* > 0.05), they were dropped from the model. Total mass and fruit mass were natural log trans-formed to improve the distribution of residuals and increase the homogeneity of variance. A Gaussian distribution and identity link function were used in all analyses. Generalized linear models were carried out using R software [66]. Tukey’s *post-hoc* test was performed using the package *multcomp* (ver. 1.4–1).

In the replacement experiment, actual RYs were compared to their expected values [0.25 (or 0.75) for species *i* (or *j*) at 2*i*:6*j* proportion, 0.50 for species *i* and *j* at 4*i*:4*j*, and 0.75 (or 0.25) for species *i* (or *j*) at 6*i*:2*j*] and actual RYTs to their expected value (1.0) at each proportion by *t* tests. Mean aggressivity values between invasive and non-invasive congeners were compared by Welch’s two sample *t*-tests for each phylogenetic group.

## Results

### Heterocarpy experiment

**Fig 2:**
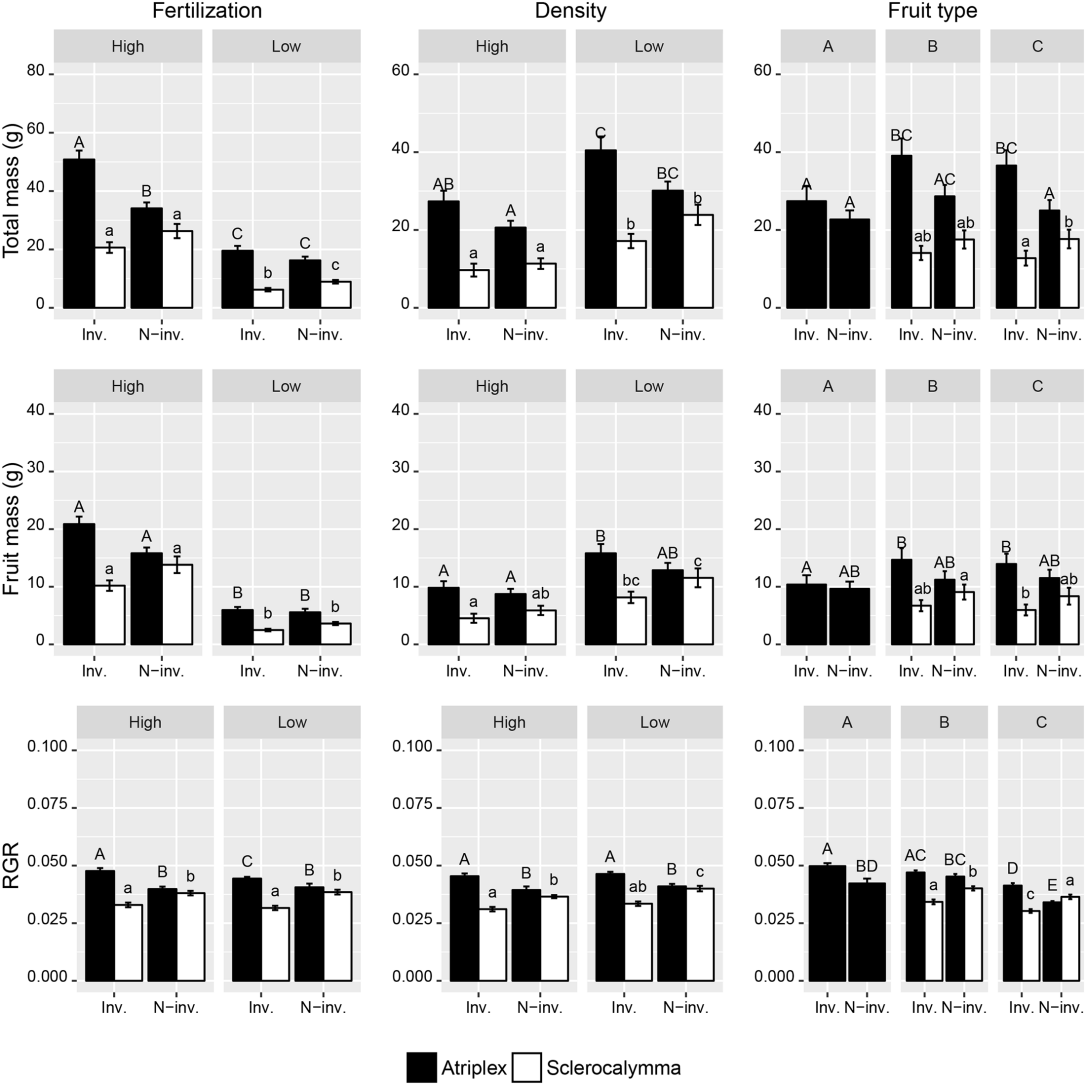
Results of the heterocarpy experiment. Total mass, fruit mass and relative growth rate (RGR) of invasive (Inv.) and non-invasive (N-inv.) *Atriplex* species pairs representing two different sections, i.e. sect. Atriplex (*A*. *sagittata* and *A*. *hortensis*) and sect. Sclerocalymma (*A*. *tatarica* and *A*. *rosea*), compared under different fertilization levels, population densities and for plants growing from different types of fruits (i.e. A, B and C, calculated across different fertilization levels and population densities). Bars represent means ± *SE*; those bearing the same letter did not differ significantly (Tukey *post-hoc* test, *P* ≤ 0.05).

The generalized linear model showed no differences in total mass, fruit mass and RGR between invasive and non-invasive species (Table 2). This resulted from contradictory trait responses between invasive and non-invasive species of individual sections (Fig 2). Within the section Atriplex the invasive species *Atriplex sagittata* performed better than the non-invasive species *A*. *hortensis* in total mass and RGR. Within the section Sclerocalymma, however, the non-invasive *A*. *rosea* reached higher values of total mass and RGR than the invasive species *A*. *tatarica* (Fig 2).

**Table 2:** Effects of section, invasive pair, fruit type and treatment (fertilization and population density) on total mass, fruit mass and relative growth rate tested using generalized linear models in the heterocarpy experiment.

Source		Total mass			Fruit mass			Relative growth rate		
	DF	MS	*F*-ratio	*P*	MS	*F*-ratio	*P*	MS	*F*-ratio	*P*
Section (S)	1	32.093	106.042	***	14.068	46.5985	***	0.00445	102.671	***
Invasive(Pair) (I)	1	0.025	0.0830	NS	0.127	0.4196	NS	0.00004	1.0319	NS
Fertilization (F)	1	61.033	201.670	***	84.423	279.650	***	0.00005	1.0879	NS
Density (D)	1	22.298	73.6800	***	16.118	53.3904	***	0.00032	7.3958	**
Fruit type (Fruit)	2	3.576	5.9081	**	2.758	4.5674	*	0.00297	34.3051	***
S × F	1	0.333	1.1009	NS	0.030	0.0977	NS	0.00000	0.1735	NS
S × D	1	1.613	5.3293	*	0.396	1.3123	NS	0.00004	1.1417	NS
I × F	1	0.687	2.2694	NS	0.381	1.2629	NS	0.00018	4.1878	*
I × D	1	0.033	0.1086	NS	0.306	1.0127	NS	0.00002	0.3885	NS
S × Fruit	1	0.009	0.0294	NS	0.785	2.6010	NS	0.00032	7.3937	**
I × Fruit	2	0.033	0.0549	NS	0.052	0.0854	NS	0.00096	11.0150	***

* *P* < 0.05;

** *P* < 0.01;

*** *P* < 0.001;

NS = non significant.

Differences in species performance were manifested through plants growing from the ecologically most contrasting fruit types specifically for each section. In the section Atriplex, species trait differences were positively related to type A fruit (in the case of RGR) or type C fruit (in the case of total mass and RGR) (Fig 2), in no case were they related to type B fruit. In the section Sclerocalymma, species differences were found between plants arising from either type B fruit (RGR) or type C fruit (total mass and RGR) (Fig 2).

### Replacement experiment

Actual RYs of *A*. *sagittata* were significantly lower than expected at two of three proportions when grown with *A*. *hortensis* (*P* ≤ 0.034) (Fig 1). While the RY of *A*. *rosea* was lower than expected when the species were grown at equal proportions (*P* = 0.0003), *A*. *tatarica* reached higher relative yield than expected when it grew under high population densities of *A*. *rosea* (*P* = 0.003). RYT was not significantly different from 1.0 at all proportions in these three mixture combinations for both species pairs (*P* ≥ 0.107). Mean aggressivity differed significantly between the species of both pairs (Table 3). The aggressivity of the invasive *Atriplex sagittata* was significantly lower (*P* = 0.00002) than that of the non-invasive *A*. *hortensis*, but the aggressivity of the invasive *A*. *tatarica* was higher (*P* = 0.0009) than the non-invasive *A*. *rosea*.

**Table 3:** Aggressivity coefficient (mean±*SE*) for individual *Atriplex* species pairs, i.e. *A*. *sagittata—A*. *hortensis* and *A*. *tatarica—A*. *rosea*, when grown with each other.

Section	Species	Aggressivity coefficient
Atriplex	*A*. *sagittata*	–0.627±0.178 ***
	*A*. *hortensis*	0.627±0.178
Sclerocalymma	*A*. *tatarica*	0.348±0.135 ***
	*A*. *rosea*	–0.348±0.135

The significance values were obtained by Welch two sample *t*-tests.

*** *P* < 0.001.

## Discussion

We analysed several life-history and functional traits of two congeneric pairs of invasive and non-invasive heterocarpic *Atriplex* species grown under different levels of nutrient supply and population density and evaluated the extent of resource partitioning between congeners. Our data support the ideas that (i) species invasiveness involves rather complex interactions of traits and is attributable to environmental and biotic factors that are resistant to generalization [11,17,18] and that (ii) heterocarpy is an important plant strategy that determines the performance of species in highly disturbed anthropogenic landscapes; in some cases, it may even contribute to species’ invasion success [43,45–47].

### Traits related to invasiveness in *Atriplex* species

Analyses of traits indicated higher performance of invasive species relative to its non-invasive congener within the section Atriplex and showed that the invasive *Atriplex sagittata* had a higher relative growth rate on average under both fertilization levels and population densities compared to the non-invasive *A*. *hortensis*. Relative growth rate was positively associated with plant performance and higher photosynthetic capacity of plants [67]. The success of plant invaders has often been attributed to their capacity for fast growth [4,14,15]. Particularly in some environments, such as in disturbed and resource-rich habitats, invading fast-growing plants exploit available resources more efficiently than slow-growing natives [10,14,15].

We found that under higher fertilization levels the invasive *Atriplex sagittata* had a greater total mass than the non-invasive *A*. *hortensis*. *Atriplex sagittata* typically colonizes various human-made habitats characterized by a high level of disturbance, low moisture and high concentrations of nitrogen [68]. Thus, the high relative growth rate and ability to maximize biomass in nutrient-rich environments predispose it to be a successful invader. This is in accordance with predictions of the fluctuating resource hypothesis, which postulated that the species’ invasion success is facilitated by its ability to acquire resources under high resource availability resulting from a disturbance [19]. Compared to *A*. *sagittata*, its non-invasive congener *A*. *hortensis* turned out to be the better competitor when the species were grown together in the replacement series. Although this predisposes the species to overall success, there are likely some constrains which impede its escape from cultivation. One of them may be a generally low level of dormancy accompanied by high seed germinability under low temperatures [43]. In the climatic conditions of Central Europe, the majority of seeds seem to germinate in autumn and seedlings probably do not survive winter, or the majority of seeds germinate in spring and only a limited number of seeds get stored in the seed bank [43]. So, even though *A*. *hortensis* is a heterocarpic species that should follow a so-called ‘bet-hedging’ strategy, its seeds are in fact ecologically equivalent and do not store part of the progeny until the next generations. Population regeneration following population failure is not effectively buffered by seeds accumulated in a seed bank, as in the case of *A*. *sagittata*.

Surprisingly, within the section Sclerocalymma, the non-invasive *Atriplex rosea* reached higher values of some traits than the invasive *A*. *tatarica*. It had a higher relative growth rate on average across both treatments and higher total mass under low fertilization levels compared to the invasive *A*. *tatarica*. Although *A*. *rosea* seemed to have the potential to be more invasive on the basis of an analysis of life-history traits, it has currently disappeared from Central Europe. However, it was a relatively common species at the beginning of the 20^th^ century, when it typically occurred on rural farms and in villages with numerous fowl runs, which are rare today [44]. Today, it is invasive in certain parts of southwestern North America, where it is a widely established weedy species of disturbed sites, often occurring in riparian habitats, barnyards, animal bed grounds and along roadsides [69]. Its congener *A*. *tatarica*, which in the past occupied similar habitats as *A*. *rosea* [44], is currently spreading north along roads also in areas that are quite cold for species with C_4_ photosynthesis such as central and northern Poland and Germany [70,71]. Both species of the section Sclerocalymma are salt-tolerant weak competitors inhabiting open habitats where competitive pressure of other plant species is reduced to a minimum. This adaptation might be crucial mainly in cases of spreading along roads treated with salt in winter. After we planted both species together in the replacement series, we observed that *A*. *tatarica* reduced the final biomass of *A*. *rosea* and proved to be a stronger competitor. Assuming there are no differences in habitat preferences or germination requirements [43] between the two species, the competitive superiority of *A*. *tatarica* could favour it over *A*. *rosea* in alternate habitats and perhaps explain its current progressive tendency to spread in parts of Central Europe.

To sum up, even though we compared two pairs of phylogenetically closely related invasive and non-invasive species that largely share the same reproductive biology, growth form and habitat preferences, the results concerning traits shared by invasive species are inconclusive. The fact that we did not detect any general relationship between invasive status and individual traits is particularly surprising, given how similar and in a certain sense comparable the study species are with respect to their phylogenetic proximity, life history type and growth form. We therefore conclude that even though congeners offer the best available methodical framework for comparing traits among similar species, this approach might not be suitable for identifying invasive traits because of the complexity underlying invasiveness.

### Heterocarpy

Annual representatives of the genus *Atriplex* are usually heterocarpic and follow a bet-hedging strategy, as previously documented for many *Atriplex* species [35,43–47,72–77]. Although some aspects of particularly earlier phases of the life cycle (i.e. germinability and dispersibility) were frequently addressed in previous studies, traits closely related to the performance of individuals originating from different fruit types were hardly explored at all (but see [47,78]). More extensive evidence comes from studies on species of the family Asteraceae, supporting differences in, for example, survival, growth, competitive ability, life history and demographic characteristics (see [21–23] and references therein).

Our analysis revealed similar patterns of traits for members of *Atriplex* species. Plants growing from particular fruit types differed in their allocations to plant biomass and relative growth rate, but the differences were both species- and trait-specific (Fig 2). More interestingly, we found consistent trait differences between plants growing from different fruit types, which might explain the invasion success of some heterocarpic *Atriplex* species. We showed that differences in trait characteristics positively related to species invasion success are always associated with type C fruit or sometimes with type A fruit, but never with type B fruit. This trend was conspicuous for species of the section Atriplex having three types of fruit that are ecologically differentiated. It seems that fruit types with contrasting ecological behaviours play important roles in the process of invasion or, more generally, in the spreading of any heterocarpic species in a variable environment. This may be realized through maximizing population growth (via plants growing from the highly abundant non-dormant type C fruit) and by enhancing the likelihood of establishing a new population after a major disturbance (via plants arising from the deeply dormant type A fruit). Several studies dealing with heterocarpy within the genus *Atriplex* have demonstrated its important role for overall species performance in a wide range of conditions and under intensive disturbances of human-made habitats [40,41,43,45–47]. The specific roles of different fruit types determining species success have been clarified especially in the case of *A*. *sagittata*, which has three types of fruit that differ in their germination and dispersal characteristics [43,45]. Type A (ebracteate) fruit is deeply dormant and has the lowest dispersibility. Of the two more dispersible fruit types with bracts, type B fruit exhibits a certain degree of dormancy whereas type C fruit is non-dormant [46]. While at least a portion of dormant types A and B fruits remain in the seed bank, ensuring long-term species survival in a highly disturbed environment, the most abundant non-dormant C fruits germinate immediately when conditions become favourable. Mandák and Pyšek [41], with regard to dormancy patterns, stated that the success of *A*. *sagittata* in the present Central European landscape might, at least partly, be attributed to its heterocarpy and associated plastic response to changing environmental conditions. However, comparing different species exhibiting different degrees of success in today’s countryside could disentangle the exact role of heterocarpy in this annual plant species. We have shown that heterocarpy is important, but in different phases of the plant life cycle in different species, depending on the behaviour of heterocarpic morphs. Baskin et al. [22] pointed out this knowledge gap and showed this in their review of heteromorphic species of cold deserts of northwest China. They stated that heterodiaspory is without doubt a significant adaptation in cold desert annuals. We suggest that heterocarpy in the sense of preadaptation to cold desert environments constitutes a set of complex adaptations which may predispose species to successful establishment and invasions in a wide range of habitats and under intensive disturbances typical of man-made landscapes. However, to generalize these results, studies involving more model groups with different levels of heterocarpy and invasive status should be performed.

## Conclusions

The main result of this study is that in a comparison of life history traits of two species pairs, the relative importance of heterocarpy, as opposed to other biological aspects in the current invasion success of closely related *Atriplex* species, clearly differs. Whilst differences in biomass production and growth characteristics could explain the current invasibility of *A*. *sagittata*, invasive species had surprisingly lower competitive ability. Interestingly, the differences between species were manifested in plants growing from the ecologically most differentiated fruit types. On the other hand, the invasive success of *A*. *tatarica*, which reached lower overall growth rates than its congener *A*. *rosea*, is attributable to its higher competitive ability or other, as yet unknown, biological aspects associated with its ability to occupy alternate habitats. Hence, the invasive species under study did not differ in functional traits from non-invasive ones across the sections, supporting the notion that species invasiveness involves more complex interactions of traits that may be important only in certain contexts. Specific species-habitat interactions are therefore very important for determining species invasiveness. This view is also supported by the outcomes of current invasion biology [11,17,18], pointing out that invasibility is rather resistant to generalization. Thus, the study of congeners is unlikely to reveal traits responsible for invasiveness because of the complexity underlying the whole invasion process.

## Supporting information

S1 Dataset Data from heterocarpy experiment. (XLSX)

S2 Dataset Data from replacement experiment. (XLSX)

## References

[1] Rejmánek M, Richardson DM. What attributes make some plant species more invasive? Ecology. 1996; 77: 1655–1661.

[2] Hamilton MA, Murray BR, Cadotte MW, Hose GC, Baker AC, Harris CJ, et al Life-history correlates of plant invasiveness at regional and continental scales. Ecol Lett. 2005; 8: 1066–1074.

[3] Pyšek P, Richardson DM. Traits associated with invasiveness in alien plants: Where do we stand? In: Nentwig W, editor. Biological invasions, Ecological Studies 193 Berlin & Heidelberg: Springer-Verlag; 2007 pp. 97–126.

[4] van Kleunen M, Weber E, Fischer M. A meta-analysis of trait differences between invasive and non-invasive plant species. Ecol Lett. 2010; 13: 235–245. 10.1111/j.1461-0248.2009.01418.x 20002494

[5] Groves RH, Panetta FD, Virtue JG. Weed risk assessment. Melbourne: CSIRO Publishing; 2001.

[6] Dawson W, Burslem DFRP, Hulme PE. The comparative importance of species traits and introduction characteristics in tropical plant invasions. Divers Distrib. 2011; 17: 1111–1121.

[7] Hawkes CV. Are invaders moving targets? The generality and persistence of advantages in size, reproduction, and enemy release in invasive plant species with time since introduction. Am Nat. 2007; 170: 832–843. 10.1086/522842 18171166

[8] Mason RAB, Cooke J, Moles AT, Leishman MR. Reproductive output of invasive versus native plants. Glob Ecol Biogeogr. 2008; 17: 633–640.

[9] Ordonez A, Wright IJ, Olff H. Functional differences between native and alien species: a global-scale comparison. Funct Ecol. 2010; 24: 1353–1361.

[10] Daehler CC. Performance comparisons of co-occurring native and alien invasive plants: implications for conservation and restoration. Annu Rev Ecol Evol Syst. 2003; 34: 183–211.

[11] Moles AT, Flores-Moreno H, Bonser SP, Warton DI, Helm A, Warman L, et al Invasions: the trail behind, the path ahead, and a test of a disturbing idea. J Ecol. 2012; 100: 116–127.

[12] Richards CL, Bossdorf O, Muth NZ, Gurevitch J, Pigliucci M. Jack of all trades, master of some? On the role of phenotypic plasticity in plant invasions. Ecol Lett. 2006; 9: 981–993. 10.1111/j.1461-0248.2006.00950.x 16913942

[13] Davidson AM, Jennions M, Nicotra AB. Do invasive species show higher phenotypic plasticity than native species and, if so, is it adaptive? A meta-analysis. Ecol Lett. 2011; 14: 419–431. 10.1111/j.1461-0248.2011.01596.x 21314880

[14] Burns JH. A comparison of invasive and non‐invasive dayflowers (Commelinaceae) across experimental nutrient and water gradients. Divers Distrib. 2004; 10: 387–397.

[15] González AL, Kominoski JS, Danger M, Ishida S, Iwai N, Rubach A. Can ecological stoichiometry help explain patterns of biological invasions? Oikos. 2010; 119: 779–790.

[16] Ordonez A, Olff H. Do alien plant species profit more from high resource supply than natives? A trait-based analysis. Glob Ecol Biogeogr. 2013; 22: 648–658.

[17] Kueffer C, Pyšek P, Richardson DM. Integrative invasion science: model systems, multi-site studies, focused meta-analysis and invasion syndromes. New Phytol. 2013; 200: 615–633. 10.1111/nph.12415 23879193

[18] Leffler AJ, James JJ, Monaco TA, Sheley RL. A new perspective on trait differences between native and invasive exotic plants. Ecology. 2014; 95: 298–305. 24669724 10.1890/13-0102.1

[19] Davis MA, Grime JP, Thompson K. Fluctuating resources in plant communities: a general theory of invasibility. J Ecol. 2000; 88: 528–534.

[20] Chytrý M, Jarošík V, Pyšek P, Hájek O, Knollová I, Tichý L, et al Separating habitat invasibility by alien plants from the actual level of invasion. Ecology. 2008; 89: 1541–1553. 18589519 10.1890/07-0682.1

[21] Mandák B. Seed heteromorphism and the life cycle of plants: a literature review. Preslia. 1997; 69: 129–159.

[22] Baskin JM, Lu JJ, Baskin CC, Tan DY, Wang L. Diaspore dispersal ability and degree of dormancy in heteromorphic species of cold deserts of northwest China: A review. Perspect Plant Ecol Evol Syst. 2014; 16: 93–99.

[23] Imbert E. Ecological consequences and ontogeny of seed heteromorphism. Perspect Plant Ecol Evol Syst. 2002; 5: 13–36.

[24] Cohen D. Optimizing reproduction in a randomly varying environment. J Theor Biol. 1966; 12: 119–129. 6015423 10.1016/0022-5193(66)90188-3

[25] Venable DL, Lawlor L. Delayed germination and dispersal in desert annuals: escape in space and time. Oecologia. 1980; 46: 272–282. 10.1007/BF00540137 28309684

[26] Levin SA, Cohen D, Hastings A. Dispersal strategies in patchy environments. Theor Popul Biol. 1984; 26: 165–191.

[27] Schoen DJ, Lloyd DG. The selection of cleistogamy and heteromorphic diaspores. Biol J Linn Soc Lond. 1984; 23: 303–322.

[28] Venable DL, Brown JS. The population-dynamic functions of seed dispersal. Vegetatio. 1993; 108: 31–55.

[29] Kistenmacher M, Gibson JP. Bet-hedging against larval herbivory and seed bank mortality in the evolution of heterocarpy. Am J Bot. 2016; 103: 1383–1395. 10.3732/ajb.1600078 27507839

[30] Baker GA, O'Dowd DJ. Effect of parent plant density on the production of achene types in the annual *Hypochoeris glabra*. J Ecol. 1982; 70: 201–215.

[31] Tanowitz BD, Salopek PF, Mahall BE. Differential germination of ray and disc achenes in *Hemizonia increscens* (Asteraceae). Am J Bot. 1987; 74: 303–312.

[32] Sorenson AE. Somatic polymorphism and seed dispersal. Nature. 1978; 276: 174–176.

[33] Lu JJ, Tan DY, Baskin JM, Baskin CC. Fruit and seed heteromorphism in the cold desert annual ephemeral *Diptychocarpus strictus* (Brassicaceae) and possible adaptive significance. Ann Bot 2010; 105: 999–1014. 10.1093/aob/mcq041 20348559 PMC2876001

[34] Lu JJ, Tan DY, Baskin JM, Baskin CC. Trade-offs between seed dispersal and dormancy in an amphi-basicarpic cold desert annual. Ann Bot. 2013; 112: 1815–1827. 10.1093/aob/mct240 24197752 PMC3838562

[35] Wertis BA, Ungar IA. Seed demography and seedling survival in a population of *Atriplex triangularis* Willd. Am Midl Nat. 1986; 116: 152–162.

[36] Brändel M. Dormancy and germination of heteromorphic achenes of *Bidens frondosa*. Flora. 2004; 199: 228–233.

[37] Wang L, Huang ZY, Baskin CC, Baskin JM, Dong M. Germination of dimorphic seeds of the desert annual halophyte *Suaeda aralocaspica* (Chenopodiaceae), a C4 plant without Kranz anatomy. Ann Bot. 2008; 102: 757–769. 10.1093/aob/mcn158 18772148 PMC2712381

[38] Flint SD, Palmblad IG. Germination dimorphism and developmental flexibility in the ruderal weed *Heterotheca grandiflora*. Oecologia. 1978; 36: 33–43. 10.1007/BF00344569 28309225

[39] Imbert E, Escarre J, Lepart J. Seed heteromorphism in *Crepis sancta* (Asteraceae): Performance of two morphs in different environments. Oikos. 1997; 79: 325–332.

[40] Mandák B, Pyšek P. Effects of plant density and nutrient levels on fruit polymorphism in *Atriplex sagittata*. Oecologia. 1999; 119: 63–72. 10.1007/s004420050761 28308160

[41] Mandák B, Pyšek P. How does density and nutrient stress affect allometry and fruit production in the heterocarpic species *Atriplex sagittata* (Chenopodiaceae)? Can J Bot. 1999; 77: 1106–1119.

[42] Lu JJ, Tan DY, Baskin JM, Baskin CC. Phenotypic plasticity and bet-hedging in a heterocarpic winter annual/spring ephemeral cold desert species of Brassicaceae. Oikos. 2012; 121: 357–366.

[43] Mandák B. Germination requirements of invasive and non-invasive *Atriplex* species: a comparative study. Flora. 2003b; 198: 45–54.

[44] Mandák B. Distribution of four *Atriplex* species with different degrees of invasiveness in the Czech Republic In: Child LE, Brock JH, Brundu G, Prach K, Pyšek P, Wade PM, Williamson M, editors. Plant invasions: ecological threats and management solutions. The Netherlands: Backhuys Publisher; 2003 pp. 313–328.

[45] Mandák B, Pyšek P. Fruit dispersal and seed banks in *Atriplex sagittata*: the role of heterocarpy. J Ecol. 2001; 89: 159–165.

[46] Mandák B, Pyšek P. The effects of light quality, nitrate concentration and presence of bracteoles on germination of different fruit types in the heterocarpous *Atriplex sagittata*. J Ecol. 2001; 89: 149–158.

[47] Mandák B, Pyšek P. How does seed heteromorphism influence the life history stages of *Atriplex sagittata* (Chenopodiaceae)? Flora. 2005; 200: 516–526.

[48] Muth NZ, Pigliucci M. Traits of invasives reconsidered: phenotypic comparisons of introduced invasive and introduced noninvasive plant species within two closely related clades. Am J Bot. 2006; 93: 188–196. 10.3732/ajb.93.2.188 21646179

[49] Sukhorukov AP. Zur Systematik und Chorologie der in Russland und benachbarten Staaten (in den Grenzen der ehemaligen USSR) vorkommenden *Atriplex*-Arten (Chenopodiaceae). Ann Nat Hist Mus Wien Ser B Bot Zool. 2006; 108: 307–420.

[50] Stutz HC, Chu GL, Sanderson SC. Evolutionary studies of *Atriplex*: phylogenetic relationships of *Atriplex pleiantha*. Am J Bot. 1990; 77: 364–369.

[51] Kochánková J, Mandák B. Biological flora of Central Europe: *Atriplex tatarica* L. Perspect Plant Ecol Evol Syst. 2008; 10: 217–229.

[52] Richardson DM, Pyšek P, Rejmánek M, Barbour MG, Panetta FD, West CJ. Naturalization and invasion of alien plants: concepts and definitions. Divers Distrib. 2000; 6: 93–107.

[53] Aellen P. *Atriplex* In: Hegi G, editor. Illustrierte Flora von Mitteleuropa, vol. 3/2 München: Carl Hanser Verlag; 1960 pp. 664–693.

[54] Meusel H, Jäger EJ, Weinert E. Vergleichende Chorologie der zentraleuropäischen Flora Text u. Karten, Bd. 1 Jena: VEB Fischer; 1965.

[55] Osmond CB, Björkman O, Anderson DJ. Physiological processes in Plant Ecology. Towards a synthesis with *Atriplex* Ecological Studies, vol. 36 Heidelberg: Springer; 1980.

[56] Jalas J, Suominen J. Atlas Florae Europaeae Distribution of vascular plants in Europe. II Cambridge: Cambridge University Press; 1987.

[57] Májovský J, Murín A, Feráková V, Hindáková M, Schwarzová T, Uhríková A, et al Karyotaxonomický prehľad flóry Slovenska. Bratislava: Veda; 1987.

[58] Kirschner J, Tomšovic P. *Atriplex* In: Hejný S, Slavík B, editors. Flora of the Czech Republic, vol. 2 Praha: Academia; 1990 pp. 266–280.

[59] Měsíček J, Jarolímová V. List of chromosome numbers of the Czech vascular plants. Praha: Academia; 1992.

[60] Aellen P, Akeroyd JR. *Atriplex* L In: Tutin TG, Burges NA, Chater AO, Edmondson JR, Heywood VH, Moore DM, Valentine DH, Walters SM, Webb DA, editors. Flora Europaea, Psilotaceae to Platanaceae, vol. 1 Cambridge: Cambridge University Press; 1993 pp. 135.

[61] Hendry GAF, Grime JP. Methods in comparative plant ecology A laboratory manual. London: Chapman & Hall; 1993.

[62] de Wit CT. On competition. Verslagen van landbouwkundige onderzoekingen. 1960; 66: 1–82.

[63] Harper JL. Population biology of plants. London: Academic Press; 1977.

[64] Snyder KM, Baskin JM, Baskin CC. Comparative ecology of the narrow endemic *Echinacea tennesseensis* and two geographically widespread congeners: relative competitive ability and growth characteristics. Int J Plant Sci. 1994; 155: 57–65.

[65] Walck JL, Baskin JM, Baskin CC. Relative competitive abilities and growth characteristics of a narrowly endemic and a geographically widespread *Solidago* species (Asteraceae). Am J Bot. 1999; 86: 820–828. 10371724

[66] R Development Core Team. R: a language and environment for statistical computing. Austria: Vienna; 2015.

[67] Wright IJ, Reich PB, Westoby M, Ackerly DD, Baruch Z, Bongers F, et al The worldwide leaf economics spectrum. Nature. 2004; 428: 821–827. 10.1038/nature02403 15103368

[68] Mandák B, Pyšek P. History of the spread and habitat preferences of *Atriplex sagittata* (Chenopodiaceae) in the Czech Republic In: Starfinger U, Edwards K, Kowarik I, Williamson M, editors. Plant Invasions: Ecological mechanisms and human responses. The Netherlands: Backhuys Publishers, Leiden; 1998 pp. 209–224.

[69] Khan MA, Gul B, Weber DJ. Temperature and high salinity effects in germinating dimorphic seeds of *Atriplex rosea*. West N Am Nat. 2004; 64: 193–201.

[70] Zündorf HJ, Günther KF, Korsch H, Westhus W. Flora von Thüringen. Jena: Weissdorn-Verlag; 2006.

[71] Jarzyna I, Malecka K, Panufnik-Medrzycka D, Medrzycki P. Dynamics and occurrence patterns of the tatarian orache *Atriplex tatarica* L. (Chenopodiaceae) at the roadsides in Warsaw, Poland. Acta Soc Bot Pol Pol Tow Bot. 2010; 79: 249–254.

[72] Beadle NCW. Studies on halophytes. I. The germination of the seeds and establishment of the seedlings of five species of *Atriplex* in Australia. Ecology. 1952; 33: 49–62.

[73] Koller D. Germination-regulating mechanisms in some desert seeds. IV. *Atriplex dimorphostegia* Kar. et Kir. Ecology. 1957; 38: 1–13.

[74] Koller D. Analysis of the dual action of white light on germination of *Atriplex dimorphostegia* (Chenopodiaceae). Israel Journal of Botany. 1970; 19: 499–516.

[75] Drake DR, Ungar IA. Effect of salinity, nitrogen and population density on the survival, growth and reproduction of *Atriplex triangularis* (Chenopodiaceae). Am J Bot. 1989; 76: 1125–1135.

[76] Li W, An P, Liu X, Khan MA, Tsuji W, Tanaka K. The effect of light, temperature and bracteoles on germination of polymorphic seeds of *Atriplex centralasiatica* Iljin under saline conditions. Seed Sci Technol. 2008; 36: 325–338.

[77] Kochánková J, Mandák B. How do population genetic parameters affect germination of the heterocarpic species *Atriplex tatarica* (Amaranthaceae)? Ann Bot. 2009; 103: 1303–1313. 10.1093/aob/mcp073 19339299 PMC2685308

[78] Ellison AM. Effect of seed dimorphism on the density-dependent dynamics of experimental populations of *Atriplex triangularis* (Chenopodiaceae). Am J Bot. 1987; 74: 1280–1288.

